# Molecular Architecture of Genetically-Tractable GABA Synapses in *C. elegans*

**DOI:** 10.3389/fnmol.2019.00304

**Published:** 2019-12-12

**Authors:** Xin Zhou, Jean-Louis Bessereau

**Affiliations:** Univ Lyon, Université Claude Bernard Lyon 1, CNRS UMR 5310, INSERM U 1217, Institut NeuroMyoGène, Lyon, France

**Keywords:** GABA_**A**_ receptor, *C. elegans*, neuromuscular junction, punctin, neuroligin

## Abstract

Inhibitory synapses represent a minority of the total chemical synapses in the mammalian brain, yet proper tuning of inhibition is fundamental to shape neuronal network properties. The neurotransmitter γ-aminobutyric acid (GABA) mediates rapid synaptic inhibition by the activation of the type A GABA receptor (GABA_A_R), a pentameric chloride channel that governs major inhibitory neuronal transduction in the nervous system. Impaired GABA transmission leads to a variety of neuropsychiatric diseases, including schizophrenia, autism, epilepsy or anxiety. From an evolutionary perspective, GABA_A_R shows remarkable conservations, and are found in all eukaryotic clades and even in bacteria and archaea. Specifically, *bona fide* GABA_A_Rs are found in the nematode *Caenorhabditis elegans*. Because of the anatomical simplicity of the nervous system and its amenability to genetic manipulations, *C. elegans* provide a powerful system to investigate the molecular and cellular biology of GABA synapses. In this mini review article, we will introduce the structure of the *C. elegans* GABAergic system and describe recent advances that have identified novel proteins controlling the localization of GABA_A_Rs at synapses. In particular, Ce-Punctin/MADD-4 is an evolutionarily-conserved extracellular matrix protein that behaves as an anterograde synaptic organizer to instruct the excitatory or inhibitory identity of postsynaptic domains.

## Introduction

Neurochemical synapses are the elementary structures that process the directional transfer of electrical signals in neural circuits. Based on their molecular composition, synapses probably emerged early during evolution before the divergence of Cnidarians and Bilaterians, more than 1.2 billion years ago (Sakarya et al., 2007; Emes and Grant, 2012). The molecular composition of the synapse shows high conservation. For example, among Bilaterians, a comparison of mouse genes encoding the postsynaptic proteome indicates that ≈45% have detectable orthologs in the ecdysozoans *Caenorhabditis elegans* or *Drosophila melanogaster* (Ryan and Grant, 2009). Although synapses were further diversified in the chordate lineage, it is possible to interrogate the general organization and function of chemical synapses in simple invertebrate organisms, and thereby take advantage of their ease of manipulation and the power of their genetic toolkits. In this mini review article, we outline how this strategy was successful in the nematode *C. elegans* to identify a novel organizer of inhibitory γ-aminobutyric acid (GABA)ergic synapses.

*C. elegans* is an anatomically-simple, 1 mm-long, non-parasitic nematode. Stereotyped divisions of the zygote, in combination with fixed programmed cell-death events, generate 959 somatic cells in the adult hermaphrodite and 1,033 in the adult male. The adult hermaphrodite contains 302 neurons, most of which are morphologically simple, extending only a few unbranched neurites. The connectivity of the *C. elegans* nervous system was reconstructed in the 1970s from serial EM sections (White et al., 1986). Connectivity is relatively sparse since the entire network contains less than 10,000 chemical synapses, including 1,500 neuromuscular junctions (NMJs), and about 800 gap junctions. Based on the reconstruction of few independent specimens and the visualization of specific synapses with fluorescent markers, the overall connectivity of the system appears strikingly reproducible among individuals, yet data are currently being generated using modern connectomic techniques to get a better sense of interindividual variability with single-synapse resolution (Mulcahy et al., 2018; Cook et al., 2019). This anatomical simplicity contrasts with the complexity of the molecular repertoire expressed in the nervous system. Although *C. elegans* contains 10^8^ times fewer neurons than humans, its genome contains about 22,000 genes, which is very comparable with the human gene content. All classes of neurotransmitter systems found in mammals are present within *C. elegans* (Hobert, 2018), with a remarkable diversity of peptidergic transmission and the expansion of some receptor families, such as nicotinic and olfactory receptors. Specifically, the machinery to synthesize, release and sense the neurotransmitter GABA is remarkably conserved within mammals (Schuske et al., 2004).

## GABAergic Neurotransmission in *C. elegans*

Early mapping of the GABAergic system by anti-GABA immunostaining identified 26 neurons in the *C. elegans* nervous system: 19 motoneurons (D-class) that establish NMJs on body-wall muscles, four motoneurons (RMEs) that control head muscles, two neurons (AVL and DVB) that innervate intestinal muscles and the interneuron RIS (McIntire et al., 1993b). A recent study identified 10 additional GABA-positive neurons, out of which three express the glutamic acid decarboxylase (GAD)/UNC-25, while the others might accumulate GABA by re-uptake using the plasma membrane transporter GAT/SNF-11 or some uncharacterized mechanisms (Gendrel et al., 2016).

**Figure 1:**
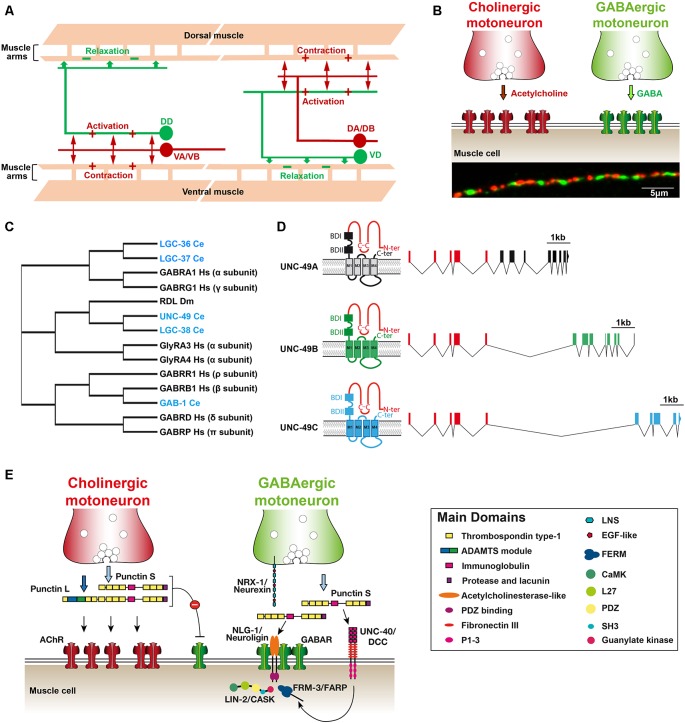
**(A)** Schematic organization of the *C. elegans* neuromuscular network. Mononucleated body-wall muscle cells on the ventral and dorsal sides of the worm extend ≈5 muscle arms to contact the axon of cholinergic (red) and γ-aminobutyric acid (GABA)ergic (green) motoneurons along the ventral and dorsal nerve cords, respectively. Cholinergic neurons (VA/VB and DA/DB) form dyadic synapses activating muscle cells and GABAergic motoneurons (DD and VD) that form inhibitory neuromuscular junctions (NMJs) on opposite muscle cells. **(B)** Distribution of excitatory and inhibitory NMJs along the ventral nerve cord. Upper panel: a schematic drawing showing that each muscle cell receives both cholinergic and GABAergic inputs. Lower panel: immunostaining of cholinergic boutons (anti-UNC-17/VAChT; red) and GABA_A_Rs (anti-UNC-49; green) at the dorsal nerve cord. **(C)** Cladogram showing the phylogenic relationships of the *C. elegans* genes encoding GABA_A_ receptor subunits (blue). The tree was adapted from Tsang et al. (2007) and Gendrel et al. (2016). Dm, Drosophila melanogaster; Hs, Homo sapiens. **(D)** Schematic structure of the *unc-49* locus encoding the GABA_A_R present at inhibitory NMJs (adapted from Bamber et al., 1999). The locus generates three distinct subunits by alternative splicing. The first five exons encode most of the extracellular N-terminal, which is common to the three subunits (red). Alternative splicing of 3′ blocks of exons encode the C-terminal part of the **A,B** and **C** subunits (black, green and blue, respectively). Putative GABA binding sites (BD) and transmembrane segments are distinct between the different subunits. **(E)** Working model of GABA_A_R clustering at NMJ in *C. elegans*. See the main text for discussion of the model. ADAMTS, a disintegrin and metalloproteinase with thrombospondin; P1-3, protein binding domain 1, 2 and 3; LNS, laminin-neurexin/sex hormone-binding globulin; EGF, epidermal growth factor; FERM, (4.1, ezrin, radixin, moesin) family; SH3, src homology 3 domain; FARP, FERM, ARH/RhoGEF and pleckstrin domain protein; CASK, calcium/calmodulin-dependent serine protein kinase.

The prominent phenotype caused by impairing GABA neurotransmission in *C. elegans* is an abnormal locomotion. Unlike mammals, *C. elegans* body-wall muscles receive both excitatory input from cholinergic motoneurons and inhibitory input from GABAergic motoneurons. When a cholinergic motoneuron releases acetylcholine (ACh), it triggers both muscle contraction and the activation of a downstream GABAergic motoneuron that projects to the opposite muscles, causing their relaxation (Figure 1A). This ensures local out of phase dorsal/ventral contraction/relaxation, the elementary component of sinusoidal locomotion (Jorgensen and Nonet, 1995). Laser ablation of GABAergic motoneurons causes a specific “shrinker” phenotype due to concomitant hyper contraction of both ventral and dorsal muscles when animals try to move backward. Similarly, RME motoneurons relax head muscles during foraging and impairment of GABA neurotransmission impacts head movements. By contrast, ablation of the AVL and DVB neurons causes a “constipated” phenotype because these neurons directly activate (rather than inhibit) the enteric muscles required for expulsion of the intestinal content (McIntire et al., 1993b). GABA-dependent excitation depends on EXP-1, a GABA-sensitive cation channel with the hallmarks of the Cys-loop receptor superfamily (Thomas, 1990; Beg and Jorgensen, 2003).

The “shrinker” phenotype was used in genetic screens as a proxy to recognize “Uncoordinated” (*unc-*) mutants with impaired GABA neurotransmission among the initial collection of mutants isolated by *Sydney Brenner* (Brenner, 1974; Hodgkin, 1983; McIntire et al., 1993a). These included mutants in *unc-25*, the single gene encoding the GABA-synthetizing enzyme GAD, *unc-47*, the first gene identified in any species to encode the vesicular GABA Transporter vGAT (McIntire et al., 1997), and *unc-49*, which codes for the GABA_A_ receptors present at NMJs (Bamber et al., 1999). Interestingly, complete inactivation of GABAergic neurotransmission produces viable mutants that can reproduce under laboratory conditions.

In addition to UNC-49, the *C. elegans* genome encodes three canonical GABA_A_ receptor subunits, the two alpha-subunit type LGC-36 and LGC-37 and the beta-subunit GAB-1, that are orthologous to GABA_A_R subunits in mammals (Tsang et al., 2007). UNC-49 and the related receptor LGC-38 are phylogenetically closer to the Drosophila receptor RDL (Figure 1C). Moreover, there are at least two additional *bona* *fide* ionotropic GABA receptors, EXP-1 and LGC-35, that are permeable to cations due to specific amino-acid composition of the channel selectivity filter (Beg and Jorgensen, 2003; Jobson et al., 2015). Of the 118 anatomically defined neuron classes of the *C. elegans* hermaphrodite, 47 neuron classes are innervated by GABAergic neurons (White et al., 1986). Twenty one of these neuron classes express at least one of the aforementioned receptors based on transcriptional reporters. The apparent inability to detect GABA_A_R expression in the rest of the GABA-innervated neurons might be due to technical limitations. However, it is also likely that additional GABA receptors remain to be characterized because the *C. elegans* genome contains up to 39 GABA/Glycine receptor-like genes, including Glutamate- and Acetylcholine-gated anion channels (Jones and Sattelle, 2008; Hobert, 2018). Finally, *C. elegans* expresses two metabotropic GABA receptors for which a comprehensive expression pattern remains to be described (Dittman and Kaplan, 2008; Schultheis et al., 2011). Interestingly, a number of neurons that do not receive direct GABAergic inputs still express GABA_A_ receptors. These receptors may mediate GABA spillover transmission as demonstrated for LGC-35, which activates cholinergic motoneurons when GABA is released by GABAergic motoneurons (Jobson et al., 2015). Notably, our knowledge of the roles of GABA_A_Rs beyond the NMJ in *C. elegans* is rudimentary since the cellular localization and function of every canonical GABA_A_Rs still remains to be characterized.

## The GABA_ergic_ Neuromuscular Junction, A Genetically-Tractable Model of Inhibitory Synapse

Paradoxically, the best characterized inhibitory synapse in *C. elegans* is the GABAergic NMJ, which might relate more closely to neuro-neuronal synapses than to “standard” NMJs. Invertebrates or Drosophila, motoneurons establish a single NMJ with myofibers containing hundreds to thousands of nuclei. This differs from neuronal innervation, where a single neuron typically receives thousands of excitatory and inhibitory inputs, building a mosaic of specialized domains concentrating receptors to match presynaptic inputs. Interestingly, the anatomical organization of the *C. elegans* neuromuscular system provides a means to interrogate a number of questions which may more closely relate to the innervation of vertebrate neurons.

First, *C. elegans* body-wall muscle cells do not fuse and remain mononucleated. Second, they send dendrite-like extensions that contact and extend along the motoneurons that run in the ventral and dorsal cords and form “en-passant” synapses. Third, as presented above, each muscle cell receives both excitatory cholinergic and inhibitory GABAergic inputs from distinct classes of motoneurons (Figures 1A,B). Based on functional (Liu et al., 2007) and EM data (White et al., 1986), each synaptic bouton likely activates receptors present on more than one postsynaptic muscle arm facing the presynaptic active zone. Hence, the *C. elegans* neuromuscular arrangement represents a very simple poly-neuronal innervation system. Specifically, it can be used to interrogate how specific compartments are built on the plasma membrane to concentrate on different neurotransmitter receptors in front of the corresponding neurotransmitter release sites.

*C. elegans* development is fast. Fourteen hours after fertilization, eggs hatch as the first larval stage. Development then proceeds through four larval stages (called L1 to L4), each separated by a molt, and reach adulthood within 2.5 days at 20°C. At hatching, only six dorsal D-class (DD) motoneurons have been generated and innervate ventral body-wall muscles. The 13 ventral D-class (VD) motoneurons differentiate during the first larval stage. DD neurons rewire at the end of the L1 stage to innervate dorsal muscles while VD neurons innervate ventral muscles (White et al., 1978; Kurup and Jin, 2015). Although the adult is about 10× bigger than L1 larvae, the number of inhibitory NMJs does not increase. Rather, additional active zones form in presynaptic boutons to scale up inhibition (Yeh et al., 2005). Because of the relatively sparse distribution of presynaptic boutons and their highly reproducible patterns across animals, forward genetic screens were successful in identifying mutants with abnormal synapses using fluorescently-tagged presynaptic proteins expressed in GABA motoneurons. These screens were extremely powerful and identified multiple proteins required for the general organization of active zones in neurons such as SYD-2/Liprin, SYD-1, and RPM-1, a founding member of the PHR (Phr1/MYCBP2, highwire and RPM-1) family of proteins (Zhen and Jin, 1999; Zhen et al., 2000; Hallam et al., 2002). However, these screens did not identify proteins specifically involved in the differentiation of inhibitory synapses.

The UNC-49 GABAA receptors are generated from a single complex locus, which generates at least three different subunits (A, B and C) by alternative splicing (Figure 1D). A block of exons encodes most of the extracellular N-terminal domain, which is shared by all subunits, while exons coding for transmembrane regions is specific to each subunit. In *Xenopus* oocytes, functional GABA receptors can be reconstituted by expressing the B-subunit either alone or in combination with the C-subunit (Bamber et al., 1999). UNC-49B and UNC-49B/C have distinct pharmacology. The positive allosteric regulator diazepam, instead of activating GABA_A_Rs, inhibits the GABA-evoked UNC-49B/C current while it has no obvious effect on the UNC-49B homomer. Neurosteroids such as pregnenolone sulfate, that enhances GABA-evoked currents in mammals, have a strong inhibitory effect on the UNC-49B receptor and much weaker effects on UNC-49-B/C. UNC-49B homomers were also found to be sensitive to the broadly-active inhibitor picrotoxin, while UNC-49B/C heteromers are resistant to it (Bamber et al., 2003).

The development of a dissection technique for adult *C. elegans* enabled stable, whole-cell voltage-clamp recording from ventral medial muscle cells and gave access to native GABA_A_Rs (Richmond and Jorgensen, 1999). Spontaneous GABAergic synaptic currents can be isolated either after the pharmacological block of AChRs (Richmond et al., 1999) or by using recording solutions that discriminate excitatory and inhibitory postsynaptic currents (Vashlishan et al., 2008). The total amount of GABA_A_R present at the muscle cell surface is usually probed by measuring the response to pressure-application of the general agonist muscimol, and the synaptic pool can be activated after optogenetic stimulation of GABA motoneurons (Liewald et al., 2008). *in vivo* recordings and the pharmacological analyses of endogenous GABA_A_Rs indicate that they are likely composed of UNC-49B/C heteromers.

UNC-49 GABA_A_ receptors are clustered in register with presynaptic GABAergic boutons (Figure 1B). Clustering depends on presynaptic innervation and occurs concomitantly with presynaptic differentiation based on the visualization of fluorescently-tagged synaptic proteins (Gally and Bessereau, 2003). However, a detailed longitudinal analysis is still missing to ascertain the precise temporal relationship between pre- and postsynaptic differentiation. Remarkably, in mutants that do not synthesize GABA, both pre- and postsynaptic structures are indistinguishable from wild type, demonstrating that “inhibitory” synapses differentiate in the absence of neurotransmission (Gally and Bessereau, 2003). This situation is not unique since various synaptic types were also reported to differentiate in mammalian cell cultures and in mice in the absence of neurotransmitter release (Misgeld et al., 2002; Varoqueaux et al., 2002; Sigler et al., 2017). Fluorescently-tagged UNC-49 receptors remain functional. Again, because the distribution of these receptors is stereotyped, screens for mutants with abnormal fluorescence distribution identified factors specifically required for the differentiation and organization of GABA NMJs, as described below.

## Molecules Basis of UNC-49 Receptor Clustering in *C. elegans*

In mammalian neurons, GABA_A_Rs clustering mostly relies on the scaffolding protein gephyrin that is hypothesized to form an intracellular lattice providing anchoring sites for synaptic GABA_A_Rs (Fritschy et al., 2008; Tyagarajan and Fritschy, 2014). Collybistin, a GTP/GDP exchange factor (GEF) interacts with the synaptic adhesion protein Neuroligin-2 and promotes the clustering of gephyrin and GABA_A_Rs (Kins et al., 2000; Tyagarajan et al., 2011). Although Gephyrin acts as a prominent player for GABA_A_Rs synaptic clustering, gephyrin-independent GABA_A_R clustering can occur and the requirement of gephyrin for GABA_A_R clustering is dependent on neuronal and synapse type (Kneussel et al., 2001; Tretter et al., 2012). Interestingly, gephyrin and collybistin are not conserved in *C. elegans*, giving access to a different molecular organization for GABA_A_R clustering.

## Ce-Punctin/MADD-4

The postsynaptic assembly of cholinergic and GABAergic NMJs in *C. elegans* relies on a recently-identified anterograde synaptic organizer Ce-Punctin/MADD-4 (Muscle Arm Development Defective-4). Ce-Punctin belongs to a family of poorly characterized extracellular matrix proteins, the ADAMTS-like proteins, that contain multiple thrombospondin-repeat, immunoglobulin, and structurally-unsolved domains (Apte, 2009). There are two *madd-4* orthologs in vertebrates, *Punctin1/ADAMTSL1* and *Punctin2/ADAMTSL3*. The precise function of these genes is unknown. However, a variant of *Punctin1* was recently shown to cause a complex phenotype including congenital glaucoma, craniofacial and other systemic features (Hendee et al., 2017). *Punctin2* is expressed in the brain and was identified as a susceptibility gene for schizophrenia (Dow et al., 2011). Whether these proteins are involved in synaptic organization has not been determined.

* Ce-punctin* generates long (Punctin L) and short (Punctin S) isoforms by the use of alternative promoters. Punctin S was initially found to attract muscle arm growth and be required for midline-oriented guidance in *C. elegans* (Seetharaman et al., 2011). The role of *Ce-punctin* in synaptic organization was subsequently identified in a visual screen for mutants with abnormal positioning of fluorescently-tagged AChRs at NMJs (Pinan-Lucarré et al., 2014). Punctin L is only expressed in cholinergic motoneurons and secreted in the synaptic cleft where it triggers postsynaptic clustering of AChRs. Punctin S is expressed in both cholinergic and GABAergic neurons (Figure 1E). At cholinergic synapses, Punctin S inhibits the attraction of GABA_A_Rs by Punctin L, possibly following heterodimerization of the L and S isoforms. At GABAergic synapses, Punctin S promotes the clustering of GABA_A_Rs in front of presynaptic GABA boutons. Genetic inactivation of Punctin S does not alter presynaptic GABA boutons, but GABA_A_Rs relocalize at cholinergic synapses. Conversely, forced expression of Punctin L in GABAergic motoneurons in a *punctin* null mutant triggers the colocalization of AChRs and GABA_A_Rs opposed to GABAergic boutons (Pinan-Lucarré et al., 2014). These results demonstrated that the identity of pre- and post-synaptic domains can be genetically uncoupled *in vivo*.

Interestingly, the expression of Punctin is under direct regulation of the transcription factors that specify the terminal identity of motoneurons. The phylogenetically conserved transcription factor UNC-3 controls the expression of numerous genes required for the cholinergic neurotransmission. It also directly activates the transcription of *punctin* *L* and *S* isoforms in cholinergic motoneurons (Kratsios et al., 2015). Similarly, the homeobox transcription factor UNC-30 controls the GABAergic identity of D-type motoneurons and regulates the expression of *punctin S* (P. Kratsios and O. Hobert, personal communication). Therefore, coordinated control of motoneuron identity and Punctin expression provides a means to ensure proper coupling between presynaptic identity and postsynaptic differentiation.

## NLG-1/Neuroligin

The clustering of GABA_A_Rs at *C. elegans* NMJs requires the synaptic adhesion molecule neuroligin NLG-1. Neuroligins (NLs) are evolutionary ancient proteins that are readily detected in Bilaterians (Lenfant et al., 2014). The human genome encodes 5 NLs that support trans-synaptic adhesive functions at excitatory and inhibitory synapses and contribute to postsynaptic receptor clustering (for review see Südhof, 2008). The *C. elegans* genome contains only one NL-coding gene, *nlg-1*, which is expressed in multiple types of neurons and in the muscle (Hunter et al., 2010). NLG-1 shares about 25% identity with human NLs and cannot be related to one specific paralog. However, the core protein organization is conserved between mammals and the nematode (Calahorro, 2014). Three main NLG-1 isoforms are generated by alternative splicing of exons encoding cytoplasmic domains of the protein (Calahorro et al., 2015). This splicing seems developmentally regulated but the precise complement of NLG-1 isoforms expressed in neurons and muscle and its functional relevance remains to be analyzed.

In muscle, NLG-1 is only found at GABAergic NMJs and strictly colocalizes with the UNC-49 GABA_A_Rs (Maro et al., 2015; Tu et al., 2015). Disruption of *nlg-1* causes a redistribution of the GABA_A_Rs out of the GABA receptor domains and a reduction of the frequency and amplitude of spontaneous miniature inhibitory postsynaptic currents (mIPSCs). The synaptic localization of NLG-1 depends on Punctin S, which directly binds the NLG-1 ectodomain. The intracellular moiety of NLG-1 is dispensable for its synaptic localization but is required for its ability to cluster GABA_A_Rs (Maro et al., 2015; Tu et al., 2015).

GABA motoneurons also express NRX-1, the sole ortholog of the mammalian neurexins that are presynaptic ligands of neuroligins (reviewed in Südhof, 2008). NRX-1 is present at presynaptic sites of GABAergic NMJs but is not required for the synaptic localization of NLG-1. Based on genetic evidence, NRX-1 was proposed to work in parallel with Punctin to promote the clustering of GABA_A_Rs (Maro et al., 2015). The *nrx-1* mutant used in this study no longer expressed the NRX-1 γ isoform, which was recently shown to be important for the presynaptic organization (Kurshan et al., 2018). However, most of the ectodomain of the NRX-1 α isoform potentially remained synthesized. Therefore, the positive interaction between NRX-1 and Punctin at GABA synapses remains to be further investigated in this system.

## UNC-40/DCC

At the *C. elegans* NMJ, the synaptic content of GABA_A_Rs depends on the netrin receptor UNC-40/DCC (deleted in colorectal cancer; Tu et al., 2015). This receptor has been implicated in a wide range of developmental events involving cellular migration and axonal navigation (Chan et al., 1996; Keino-Masu et al., 1996). It is a single transmembrane domain protein that does not contain any obvious catalytic domain. Upon netrin binding, UNC-40 is believed to dimerize, causing the intracellular domains to serve as a signaling platform to recruit or activate numerous downstream targets, including several signal transduction molecules that regulate cytoskeletal dynamics (for reviews see Finci et al., 2015; Boyer and Gupton, 2018).

In *C. elegans*, UNC-40 plays a specific role in the neuromuscular system. First, it promotes the growth of muscle arms (Alexander et al., 2009). At the early larval stage, the Punctin S localizes UNC-40 at the tip of the muscle arms and, together with the guidance cue UNC-6/netrin, activates UNC-40. Thus, the number of muscle arms that project to the ventral and dorsal nerve cords is drastically reduced in *unc-40* mutants. However, the number of GABAergic boutons is unaffected and NLG-1 postsynaptic clusters remain readily detected. In addition, UNC-40 controls the amount of GABA_A_Rs at synapses. In *unc-40* mutants, there is a 60% reduction of receptors at GABAergic NMJs. A constitutively-activated version of UNC-40, which only contains the intracellular moiety of UNC-40 targeted to the plasma membrane, rescues the synaptic clustering of GABA_A_Rs (Tu et al., 2015). This suggests that upon activation by Punctin, UNC-40 promotes the recruitment of GABA_A_Rs onto NLG-1 clusters.

## FRM-3/FARP and LIN-2/Cask

Recently, the intracellular proteins FRM-3 and LIN-2 were reported to regulate GABA_A_Rs at NMJs (Tong et al., 2015). FRM-3 was initially described as a band 4.1 (EPB4.1) paralog (Tong et al., 2015), but the recently annotated FRM-3B isoform appears to be the unambiguous ortholog of the mammalian FARP1 and FARP2 proteins. FARPs are able to modulate F-actin assembly and regulate neuronal development and synaptogenesis by interacting with cell-surface proteins such as SynCAM1 and class A Plexins (Cheadle and Biederer, 2012, 2014). In *C. elegans*, FRM-3 binds the intracellular loop of the UNC-49B subunit (Tong et al., 2015). It also binds LIN-2, the ortholog of CASK, a membrane-associated guanylate kinase (MAGUK) multidomain scaffolding molecule (Hata et al., 1996) that plays important role in presynaptic assembly at mammalian synapses. At GABAergic NMJs, disruption of *frm-3* or *lin-2* causes about 25% reduction of mIPSC amplitude, while pressure-applied response to the GABA_A_Rs agonist muscimol was unchanged, indicating that receptors were expressed but not properly clustered at synapses. FRM-3 and LIN-2 were proposed to stabilize GABA_A_Rs at the synapse, in parallel to a Neuroligin-dependent scaffold (Tong et al., 2015). The precise interplay between these different proteins remains to be characterized further.

## Conclusion

GABAergic inhibitory neurotransmission is an evolutionarily ancient system that has been conserved over ≈1,000 million years of evolution. Genetic analysis in *C. elegans* identified a novel anterograde synaptic organizer, Ce-Punctin, which specifies the position of post-synaptic domains by localizing neuroligin in register with synaptic boutons, and controls the number of postsynaptic receptors through the activation of UNC-40/DCC (Figure 1E). The conservation of this system still remains to be tested in mammals. Even at the *C. elegans* NMJ, several questions remain unanswered: how is Punctin secreted and confined at synapses? What are the mechanisms that differentiate Punctin function at cholinergic and GABAergic synapses? To what extent is this system regulated by synaptic activity? Most surprisingly, it is amazing to see that the cellular and molecular basis of synaptic neuro-neuronal GABA transmission in *C. elegans* remains *terra incognita*. Lots remain to be learned.

## Author Contributions

XZ and J-LB wrote the manuscript.

## Conflict of Interest

The authors declare that the research was conducted in the absence of any commercial or financial relationships that could be construed as a potential conflict of interest.
